# Hydrothermal Synthesis of Rare-Earth Modified Titania: Influence on Phase Composition, Optical Properties, and Photocatalytic Activity

**DOI:** 10.3390/ma12050713

**Published:** 2019-02-28

**Authors:** Nejc Rozman, David M. Tobaldi, Uroš Cvelbar, Harinarayanan Puliyalil, João A. Labrincha, Andraž Legat, Andrijana Sever Škapin

**Affiliations:** 1 Slovenian National Building and Civil Engineering Institute, Dimičeva 12, 1000 Ljubljana, Slovenia; nejc.rozman@zag.si (N.R.); andraz.legat@zag.si (A.L.); 2 Department of Materials and Ceramic Engineering/CICECO—Aveiro Institute of Materials, Campus Universitário de Santiago, University of Aveiro, 3810-193 Aveiro, Portugal; david.tobaldi@ua.pt (D.M.T.); jal@ua.pt (J.A.L.); 3 Department of Gaseous Electronics (F6), Jožef Stefan Institute, Jamova 39, 1000 Ljubljana, Slovenia; uros.cvelbar@guest.arnes.si; 4 IMEC, Kapeldreef 75, 3001 Leuven, Belgium; hari.org88@gmail.com

**Keywords:** TiO_2_, photocatalytic activity, rare earths, modification, visible light activity

## Abstract

In order to expand the use of titania indoor as well as to increase its overall performance, narrowing the band gap is one of the possibilities to achieve this. Modifying with rare earths (REs) has been relatively unexplored, especially the modification of rutile with rare earth cations. The aim of this study was to find the influence of the modification of TiO_2_ with rare earths on its structural, optical, morphological, and photocatalytic properties. Titania was synthesized using TiOSO_4_ as the source of titanium via hydrothermal synthesis procedure at low temperature (200 °C) and modified with selected rare earth elements, namely, Ce, La, and Gd. Structural properties of samples were determined by X-ray powder diffraction (XRD), and the phase ratio was calculated using the Rietveld method. Optical properties were analyzed by ultraviolet and visible light (UV-Vis) spectroscopy. Field emission scanning electron microscope (FE-SEM) was used to determine the morphological properties of samples and to estimate the size of primary crystals. X-ray photoelectron spectroscopy (XPS) was used to determine the chemical bonding properties of samples. Photocatalytic activity of the prepared photocatalysts as well as the titania available on the market (P25) was measured in three different setups, assessing volatile organic compound (VOC) degradation, NO_x_ abatement, and water purification. It was found out that modification with rare earth elements slows down the transformation of anatase and brookite to rutile. Whereas the unmodified sample was composed of only rutile, La- and Gd-modified samples contained anatase and rutile, and Ce-modified samples consisted of anatase, brookite, and rutile. Modification with rare earth metals has turned out to be detrimental to photocatalytic activity. In all cases, pure TiO_2_ outperformed the modified samples. Cerium-modified TiO_2_ was the least active sample, despite having a light absorption tail up to 585 nm wavelength. La- and Gd-modified samples did not show a significant shift in light absorption when compared to the pure TiO_2_ sample. The reason for the lower activity of modified samples was attributed to a greater Ti^3+^/Ti^4+^ ratio and a large amount of hydroxyl oxygen found in pure TiO_2_. All the modified samples had a smaller Ti^3+^/Ti^4+^ ratio and less hydroxyl oxygen.

## 1. Introduction

Titania is considered one of the essential materials in green chemistry and is certainly expected to significantly impact future development in sustainability [1,2]. Because of its versatility, it can be used in environmental clean-up, either water purification [3,4] or air pollutant removal [5,6], as well as a catalyst for solar hydrogen production [7,8]. Titania is also biologically inert and chemically stable and is generally considered non-toxic [9]. An additional advantage is that it is cheap and readily available [10].

On the other hand, titania has some severe limitations. Both of titania’s most important crystal forms, anatase and rutile, have a fairly wide band gap, which in turn influences its activity as a photocatalyst and therefore also its use [11,12,13]. While one of the crystal forms, anatase, with a band gap of 3.2 eV, absorbs exclusively ultraviolet (UV) irradiation, rutile shows some promise also as a visible light (Vis) active photocatalyst, due to a somewhat narrower band gap of 3.0 eV. However, as only a small percent of solar energy is represented by UV or near-UV irradiation, even rutile’s band gap is a serious limitation for the potential usage of titania [14]. In many areas of application, such as indoor air cleaning, the UV irradiation intensity is much lower than in direct sunlight. Narrowing the band gap of titania is therefore of utmost importance in order to expand its use indoors as well as to increase the overall performance, although there are many factors that influence overall photocatalytic activity besides band gap energy [1,15]. 

Band gap modification has been addressed by introducing foreign elements into the titania crystal structure. Much work has been done on modifying titania with transition metal cations, such as Fe, V, Co, Ni, Mn, Cr, Cu, Ag, W, etc. [16,17,18,19,20,21,22,23]. Promising results have also been achieved by modifying titania with non-metal elements, such as N, S, C, B, etc. [24,25,26,27,28]. Different combinations of elements for modification have also been tried. Although there are many reports on the successful enhancement of photocatalytic activity from some authors [29], other reports usually also exist from authors who found detrimental results when modifying with the same element, or a lesser improvement [30]. In general, modification of titania may induce complex interactions and the path to visible light activity is not straightforward, as it depends on many factors, such as dopant concentration and distribution within titania particles, specific surface area, crystal modification ratio, amorphous phase content and surface water, and –OH group population. Moreover, even the properties of pollutants and the immediate environment (liquid-solid or gas-solid) of pollutant removal seem to be very important as well as pollutant and photocatalyst concentration [3]. 

Despite a plethora of papers written on this topic, modification with rare earths has been relatively unexplored, especially the modification of rutile with rare earth cations. While anatase is generally considered to have higher photocatalytic activity than other phases, there have been reports claiming that rutile can match or even out-perform anatase [31,32]. On the other hand, others claim a proper ratio between the two phases is the most effective [33,34]. Our group has found rutile to be superior to anatase regarding photocatalytic activity, and we decided to pursue the rutile modification path with the goal of enhancing the photocatalytic activity both under UV irradiation and visible light [35].

In our work, we used TiOSO_4_ as a source of titanium due to its industrial use as a reagent. To improve the functional properties of the material, namely, its photocatalytic activity, we modified our powder samples with selected rare earth (RE) elements, the REs being cerium, lanthanum, and gadolinium. Hydrothermal treatment was selected as a crystallization step. The photocatalytic activity of samples was assessed in gas-solid systems, using both volatile organic compound (VOC) and NO_x_ as model pollutants, as well as in a liquid-solid system with methylene blue (MB) as a model pollutant. Activities of the synthesized powder samples were compared to the commercial benchmark Evonik P25 across the three listed methods that represent models for potential use.

## 2. Materials and Methods 

### 2.1. Sample Preparation

Titanium oxysulphate, TiOSO_4_, (Sigma-Aldrich, 29% ≥ as TiO_2_) was used as a titanium containing a reagent. A 15 wt.% amount of the TiOSO_4_ powder was put into distilled water and stirred magnetically until it turned into a transparent solution. Then, an ammonium hydroxide solution, with an ammonia content of 25% (Sigma-Aldrich, NH_4_OH) was diluted to have an ammonia content of 10%. The ammonia solution was added instantaneously to a titanium oxysulphate solution under vigorous stirring so that the pH value of the precipitated gel reached 8. The obtained amorphous gel was then filtered and rinsed several times using distilled water, so that no residual sulphate or ammonium ions remained. After rinsing, water was added to the gel, so that the TiO_2_ content was 5 wt.%. The dispersed gel was then mixed in a dissolver at 4500 rpm for at least 30 min. The obtained dispersion was transferred to a standard Teflon-lined stainless steel autoclave with a total volume of 50 mL, where it was peptized using nitric acid (HNO_3_). The molar ratio of TiO_2_ to HNO_3_ was 1:1. To dope the samples, a calculated amount of rare earth nitrate crystal hydrate (Ce(NO_3_)_3_∙6H_2_O, La (NO_3_)_3_∙6H_2_O, and Gd(NO_3_)_3_∙6H_2_O) was dissolved in water. The amount was calculated so that the modification level of the samples would be 1 mol % in the final TiO_2_ product. A 1 mL portion of the obtained solution was added to the mixture already in the autoclave and stirred for at least 15 min. Before enclosing the autoclaves, isopropyl alcohol (IPA) was added to the mixture for phase composition control. The molar ratio between IPA and TiO_2_ was 0.4:1, which corresponds to ~0.5 mL in a total of IPA. The total filling of the autoclaves was ~80%. Autoclaves were then sealed and put into a pre-heated oven at 200 °C for 18 h. 

**Figure 1:**
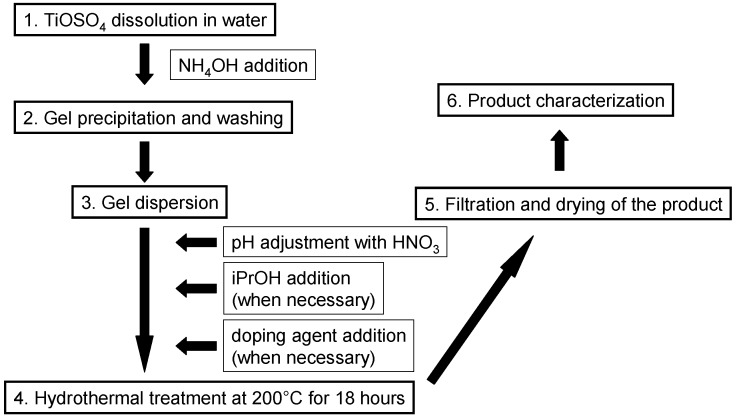
Schematic diagram of the synthesis procedure.

After the heating phase, the autoclaves were left to cool down at ambient temperature. The obtained product was filtered, rinsed with distilled water, and dried at ambient conditions (see Figure 1 for a schematic presentation of the synthesis procedure).

### 2.2. Sample Analysis

X-ray powder diffraction (XRD) analysis was used for semi-quantitative phase analysis (QPA). The data were collected using a θ/θ diffractometer (X’Pert Pro, PANalytical, Almelo, The Netherlands), equipped with a fast real time multiple strip (RTMS) detector (PIXcel 1D, PANalytical, Almelo, The Netherlands), with Cu Kα radiation (45 kV and 40 mA, 20–80° 2θ range, a virtual step scan of 0.02° 2θ and a virtual time per step of 100 s). Then, a 0.5° divergence slit, a 0.5° anti-scattering slit, 0.04 rad Soller slits, and a 15 mm copper mask were mounted in the incident beam pathway. The crystalline phases were identified via the Rietveld method, as implemented in GSAS-EXPGUI software. The method did not account for the likely presence of amorphous phase and was used solely for crystalline material phase ratio determination. The starting atomic parameters for the three TiO_2_ phases, namely, anatase, rutile, and brookite, described in the space groups *I*4_1_/*amd*, *P*4_2_/*mnm* and *Pbca*, respectively, were taken from the literature [36,37]. The instrumental contribution was obtained from the NIST SRM 660b standard (LaB_6_) and was taken into account in the refinements. The following parameters were refined: scale factors, zero-point, six coefficients of the shifted Chebyshev function to fit the background, unit cell parameters, two Lorentzian (*L*_X_ and *L*_Y_) terms, and one Gaussian term (*G*_W_, that is angle-independent) as the profile coefficients, and sample displacement effects.

We measured the optical response of the samples in UV-Vis on a Shimadzu UV 3100 (Shimadzu, Kyoto, Japan) in the range of 250–825 nm wavelength with a 0.02 nm step-size and using BaSO_4_ as a reference. The acquired diffuse reflectance spectra were converted into the absorption coefficient using the Kubelka-Munk equation:

(1) $$\alpha \approx \frac{K}{S}=\frac{\left(1-R_{\infty }\right)}{2R_{\infty }}\equiv F\left(R_{\infty }\right),$$

where *K* and *S* represent the absorption and scattering coefficients, respectively, and *R_∞_* is equal to *R_sample_/R_standard_*. The band gap was calculated by plotting the first derivative of reflectance (*dR/dλ*) versus wavelength (λ) and taking the wavelength at the maximum of this function as the energy of the band gap. This method, known as differential reflectance method, is frequently applied to calculate the energy band gap of semiconductor materials. Micrographs for size and morphology determination were taken on a Zeiss Ultra plus (Jena, Germany) field emission scanning electron microscope (FE-SEM). The X-ray photoelectron spectroscopy (XPS) analyses were carried out on a PHI-TFA XPS spectrometer produced by Physical Electronics Inc. (Chanhassen, MN, USA). The analyzed area was 0.4 mm in diameter and the analyzed depth was about 3–5 nm. This high surface sensitivity is a general characteristic of the XPS method. Sample surfaces were excited by X-ray radiation from a monochromatic Al source at a photon energy of 1486.6 eV.

### 2.3. Photocatalytic Activity Measurement

Photocatalytic activity of the samples was measured in a gas-solid and liquid-solid environment. In the gas-solid environment, two setups and two pollutants were used, namely, IPA and NO_x_. The former was used as a model VOC and the latter, as a non-organic pollutant. The model pollutant used in the liquid-solid environment was methylene blue. The setup and measurement techniques are described in more detail below.

#### 2.3.1. Photocatalytic Activity of IPA Degradation

**Figure 2:**
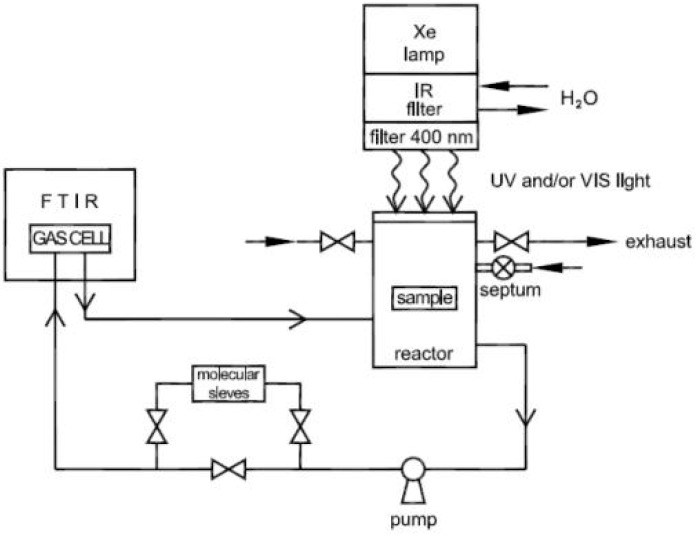
Graphical representation of the reactor system used to measure volatile organic compound (VOC) degradation over titania samples.

Photocatalytic activity of the samples was measured in a sealed gas-solid continuous flow reactor system. The system consisted of a reactor where the sample was placed, pipes, a gas pump, and a Fourier-transform infrared spectroscopy (FTIR) gas cell. Graphical representation of the reactor system used to measure VOC degradation over titania samples can be found in Figure 2. The concentrations of isopropanol, as the model pollutant, and acetone, the first product of isopropanol oxidation, were continually monitored with FTIR spectroscopy (Spectrum BXII, Perkin Elmer, Waltham, MA, USA). The method is described in detail in Marolt et al. [38] and Tobaldi et al. [39]. The method’s principle involves monitoring the degradation of the model VOC in a sealed gas reactor by FTIR spectroscopy. IPA was used as a model VOC. 

The sample was evenly spread on a small Petri dish and put into the system. Then, isopropanol was injected into the system. After the adsorption-desorption equilibrium was reached, the sample was illuminated. As the light was turned on, isopropanol concentration began to fall and acetone concentration began to rise. The reaction of isopropanol oxidation can be written as follows:

(2) $$Isopropanol\overset{k_{1}}{\to }acetone\overset{k_{2}}{\to }further\;products,$$

where *k_1_* is the reaction constant of isopropanol oxidation to acetone and *k_2_* is the reaction constant of acetone oxidation to further products. According to Munuera et al. [40] and Bickley et al. [41], the first step is a zero-order reaction, whereas the second step is a first-order reaction. Taking these assumptions into account, a combined kinetic equation of acetone formation can be written as follows:

(3) $$C_{ac}\left(t\right)\left(zero\right)=\frac{k_{1}}{k_{2}}\left(1-e^{-k_{2}t}\right),$$

where *t* is time. Equation (3) tells us that the acetone concentration curve exhibits a peak at maximum concentration. However, because *k_1_* >> *k_2_*, according to Larson et al. [42], a linear fit of the initial acetone formation kinetics curve can still be made to obtain a reliable result of the photocatalytic activity. The initial slope of the acetone concentration profile is equal to *k_1_*, which is given as a result for photocatalytic activity in VOC degradation. 

The temperature of the system was kept at 25 ± 3 °C and the relative humidity, at 22 ± 3%. The light source was a 300 W Xe lamp (Newport Oriel Instrument, Irvine, CA, USA), with an infrared filter. The lamp imitated the solar spectrum and emitted both ultraviolet (UV) and visible light (Vis). The measurement results in UV indicated that the whole spectrum of the light was used (i.e., Vis plus UV), whereas the Vis measurements indicated that a filter was used. This filter cuts out any light under 400 nm, so that in this case the samples were irradiated solely by visible light. The radiance intensity on the sample was 40 W/m^2^. Isopropanol and acetone concentrations were calculated by observing a characteristic peak at 951 cm^−1^ and 1207 cm^−1^, respectively, using a Perkin Elmer Spectrum BX spectrometer (Waltham, MA, USA).

#### 2.3.2. NO_x_ Removal Measurement

The second photocatalytic activity measurement was performed in a homemade continuous stirring tank reactor (CSTR). The method is described in detail by Lucas et al. [43]. The method’s principle involves monitoring the exhaust compounds and comparing them to the inlet gas content in the CSTR reactor. The reactor was a stainless steel cylinder with a volume of 35 L. The top of the reactor was sealed with a glass window, to allow the light to reach the photocatalyst contained inside. The light source employed was a solar lamp (Osram Ultra-Vitalux, 300 W, Munich, Germany), and the distance between it and the photocatalyst was 85 cm; the light intensity reaching the samples, measured with a radiometer (HD2302.0, Delta OHM, Padova, Italy), was found to be approximately 3.6 W·m^−2^ in the UV-A range and 25 W·m^−2^ in the visible-light range. 

Samples were prepared similarly as previously. A small amount of the photocatalyst was spread evenly on a Petri dish. The temperature of the reactor was at 27 ± 1 °C and the relative humidity was at 31%. These conditions did not change throughout the measurement. A chemiluminescence analyzer (AC-30M, Environment SA, Poissy, France) was used to measure the outlet concentration of the pollutant. The inlet gas was a mixture of synthetic air and NO_x_ (contained in separate gas cylinders). Two mass flow controllers were used to prepare a mixture, containing 0.2 ppm NO_x_, with a flow rate of 1 L/min. After the photocatalyst was put inside the reactor and the reactor was sealed, the NO_x_-containing mixture was allowed to flow through the reactor until it reached adsorption-desorption equilibrium at ~0.2 ppm. Once the desired concentration was reached, the sample was illuminated. 

The photocatalytic activity was evaluated as the ratio of removed NO_x_ from the inlet mixture. The rate of conversion was calculated according to the following equation:

(4) $$\frac{\left(NO_{x}\;conversion\right)}{\%}=\frac{{\left(NO_{x}\right)}_{0}-{\left(NO_{x}\right)}_{t}}{{\left(NO_{x}\right)}_{0}}\times 100,$$

where *(NO_x_)*_0_ is the initial NO_x_ concentration and *(NO_x_)_t_* is the NO_x_ concentration after a certain period.

#### 2.3.3. Liquid-Solid Photocatalytic Removal of Methylene Blue (MB)

A 500 mL portion of MB solution was prepared, the initial concentration of which was 5 ppm. The concentration of the photocatalyst was 0.25 g/L. The reaction took place at room temperature. The photocatalyst slurry was magnetically stirred continuously to ensure a homogeneous distribution. The lighting was provided by two germicidal lamps (PL-S 9W, Philips, Amsterdam, The Netherlands) on each side of the reactor. The intensity of UV irradiation, wavelengths between 315 and 400 nm, was measured as ~13 W·m^−2^ (measured using a radiometer HD2302.0, Delta OHM, Padova, Italy).

Sampling was done at regular intervals. Sampling consisted of taking 4 mL of the MB solution from the reactor, centrifuging for a sufficient time to separate the nanopowder from the solution, and measuring the absorbance of the solution, taking advantage of the Beer–Lambert law for the correlation between absorbance and concentration. Absorbance was measured on a spectrometer Shimadzu UV 3100 (Shimadzu, Kyoto, Japan) at 665 nm using distilled water as a reference. Photocatalytic activity was evaluated by the amount of MB degradation (seen as discoloration) and the following equation was used:

(5) $$\frac{\xi }{\%}=\frac{C_{0}-C_{t}}{C_{0}}\times 100,$$

where *C*_0_ is the initial MB concentration and *C_t_* is the concentration of MB after a certain time interval.

## 3. Results and Discussion

### 3.1. X-ray Powder Diffraction

**Figure 3:**
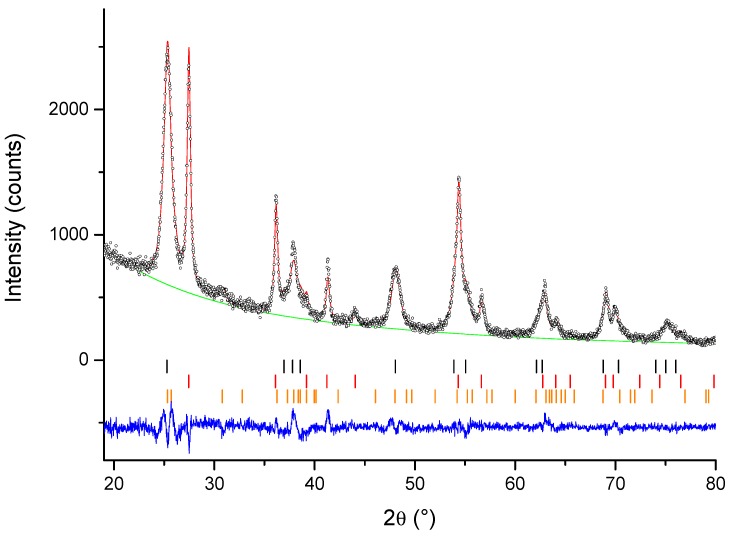
Graphic output of Rietveld refinement of sample Ce-TiO_2_. The red line represents the calculated pattern, the black open circles represents the observed pattern, and the blue line represents the difference between the observed and calculated pattern. The small vertical bars represent the positions of reflection of the three phases: black for anatase, red for rutile, and orange for brookite. The powder diffraction file numbers used were 21-1272, 21-1276, and 29-1360 for anatase, rutile, and brookite, respectively.

The phase composition results from QPA analysis are presented in Table 1 and the typical GSAS diffractogram modelling output is shown in Figure 3. The unmodified sample contains only rutile phase, whereas the modified samples are a mixture of anatase and rutile (La-TiO_2_ and Gd-TiO_2_) or a mixture of anatase, rutile, and brookite, all three of the naturally occurring titania phases (Ce-TiO_2_). While Ce modification had a significant effect on the phase ratio, rutile representing a mere 35.6 wt.%, La and Gd had a smaller effect and the sample consisted of 74.0 and 74.6 wt.% rutile, respectively, and no brookite was present. There were no additional reflections observed for the rare earth oxides (CeO_2_, La_2_O_3_, or Gd_2_O_3_), because they are most likely below the detection limit of XRD diffraction. From these results, we can see that these RE cations delay the anatase → rutile transformation, Ce^4+^ having the strongest effect. While we see that Ce^4+^ also delays brookite → rutile transformation, we cannot confirm that with La^3+^ and Gd^3+^. There have been reports of rare earth elements slowing the anatase-to-rutile transformation [44,45,46]. RE ions are larger than titanium ions (Ti^4+^ radius being 0.061 nm, Ce^3+^ radius being 0.101 nm, Ce^4+^ radius being 0.087 nm, La^3+^ radius being 1.03 nm, and Gd^3+^ radius being 0.0938 nm) and are unlikely to enter TiO_2_ crystal structure for substitutional doping. Instead, they should cluster as RE oxides on the surface of TiO_2_ particles. Since anatase-to-rutile transformation is a nucleation and growth process, where rutile nuclei form in anatase particles and grow by consuming the surrounding anatase, the addition of rare earth elements disrupts this process. Rare earth modification causes grain-boundary pinning, which delays the anatase-to-rutile transformation [45].

### 3.2. UV-Vis Spectroscopy

**Figure 4:**
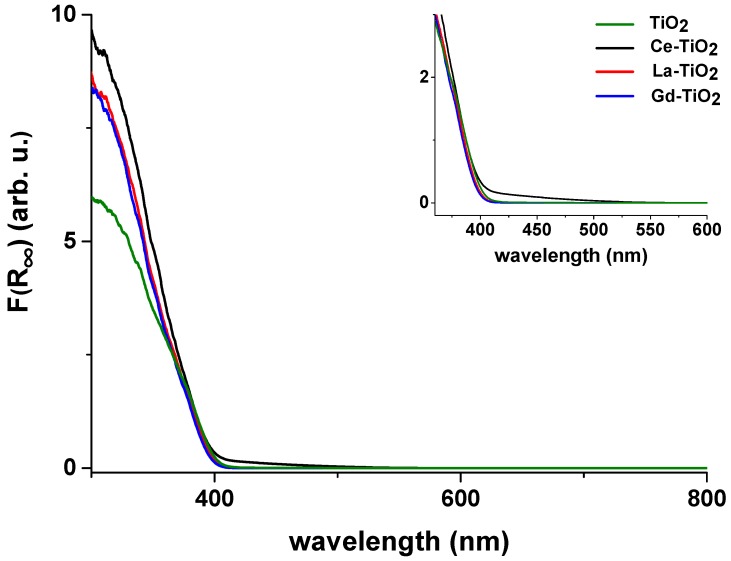
Kubelka-Munk transformation of the ultraviolet and visible light (UV-Vis) spectra of the samples. The inset in the upper right corner is a magnified graph that shows the extent of light absorption of the samples more clearly. Ce-TiO_2_ absorption tail extends to approximately 585 nm. Other samples show similar absorption characteristics. Their absorption does not seem to extend much beyond 400 nm.

The results of the transformation of the obtained UV-Vis spectra are shown in Figure 4. A single absorption band below approximately 400 nm is present in all the samples. This band is ascribed to the band-gap transition in titania [47]. There were slight differences in the absorption of the samples, the most emphasized one being the absorption of Ce-TiO_2_. The absorption of La-TiO_2_ and Gd-TiO_2_ is almost the same as that of the unmodified sample, where perhaps a very slight shift to longer wavelengths can be seen in La-TiO_2_. Unmodified TiO_2_ extends to longer wavelengths even more. A distinct tail into the visible light range absorption can be seen in sample Ce-TiO_2_, extending all the way to ~585 nm. This tail can be ascribed to the presence of CeO_2_ in the sample [48]. The calculated band gaps of the samples are given in Table 2.

### 3.3. FE-SEM 

**Figure 5:**
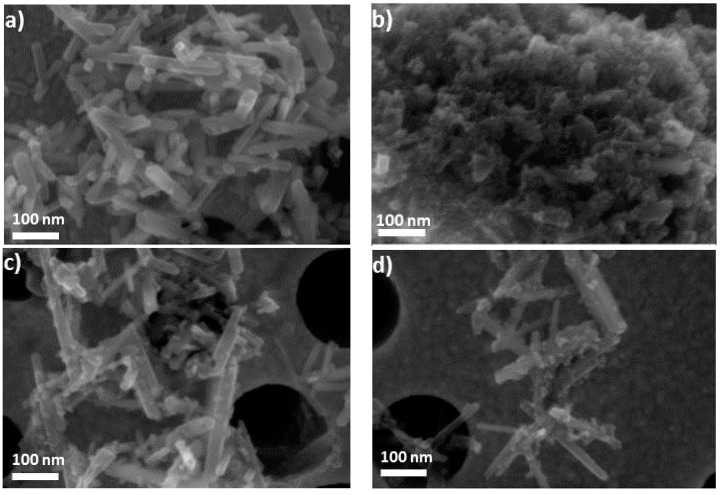
Field emission scanning electron microscope (FE-SEM) micrographs of the synthesized samples: (**a**) TiO_2_, (**b**) Ce-TiO_2_, (**c**) La-TiO_2_, and (**d**) Gd-TiO_2_. From Figure 5a we can see that rutile is composed of elongated particles. Given the prevalence of anatase in Ce-TiO_2_, we assume that the small round particles are anatase. Figure 5**c**,d shows samples La-TiO_2_ and Gd-TiO_2_, respectively, where both rutile and anatase can be observed.

Morphological analysis of samples was performed using FE-SEM as seen in Figure 5a–d. Figure 5a is a micrograph of sample TiO_2_, which is composed of rutile particles. The particles vary in size and take on elongated shape, that is, one of the three dimensions is much larger than the other two. In Figure 5b, we can see a micrograph of sample Ce-TiO_2_, composed of anatase (56 wt.%), rutile (36 wt.%), and brookite (8 wt.%). The micrograph shows some larger, elongated particles which we infer to be rutile, taking into account the micrograph in Figure 5a. There is also a large number of smaller, seemingly spherical particles. Given their prevalence and the results obtained by XRD, we assume that these are anatase particles. Brookite could not be identified from the micrographs. Figure 5c,d shows samples La-TiO_2_ and Gd-TiO_2_, respectively, where rutile particles are decorated by anatase particles.

### 3.4. XPS Analysis

**Figure 6:**
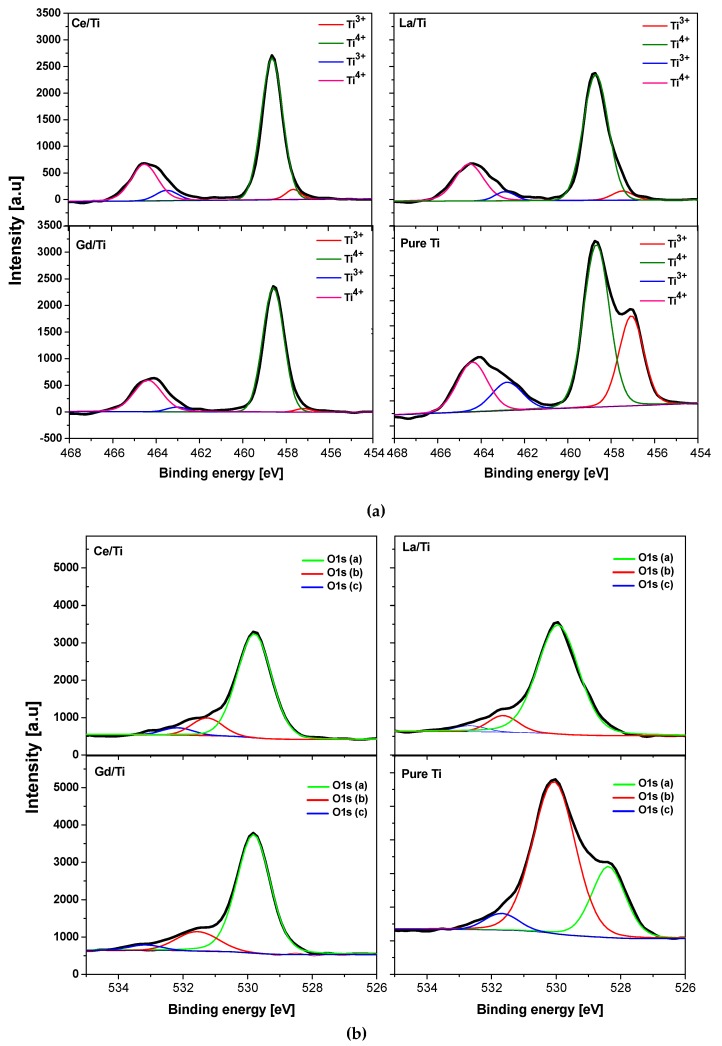
X-ray photoelectron spectroscopy (XPS) analysis results: (**a**) Ti2p de-convolution, (**b**) O1s de-convolution. We can observe that sample TiO_2_ has a larger amount of Ti^3+^ ions present, compared to other samples. The results also show that in sample TiO_2_ a significant amount of oxygen is in the form of –OH groups, whereas in other samples it exists in lattice oxygen.

The samples were analyzed by XPS in order to acquire information regarding the chemical bonding of elements on the sample surface. The obtained spectra are shown in Figure 6. Determination of the oxidation states of Ti in the composite required Ti 2p peak de-convolution. In all the modified samples, Ti^4+^ was the major chemical state of Ti. Ti^3+^ peak appears as a small shoulder at lower binding energy on both spin–orbit components of Ti 2p1/2 and Ti 2p3/2 peaks at 464.5 and 458.5 eV, respectively [49]. Surprisingly, in pure titania, the intensity of Ti^3+^ was much higher than that of the other metal-modified samples. In all the metal-modified samples, the Ti^3+^ to Ti^4+^ ratio was much lower than that of the unmodified sample and it did not differ much among the rare earth elements used. The amount of increase in the ratio of Ti^3+^/Ti^4+^ is a measure of oxygen vacancies in the material, and increased oxygen vacancy may be responsible for the enhanced catalytic activity [50]. The Ti 2p and O1s spectra of the pure titania showed a shift towards lower binding energy compared to the other modified materials. To better understand the surface chemistry of oxygen, O1s peak was de-convoluted. Typically, oxygen exists in three different chemical states in titania composites as lattice oxygen [O 1s (a)], surface hydroxyl oxygen [O 1s (b)], and adsorbed oxygen [O 1s (c)], respectively [51]. In the pure titania, the large extent of hydroxyl oxygen species was predominant, whereas rare earth element-modified samples were richer in lattice oxygen species. This is because the saturation of Ti^3+^ in the material creates a charge imbalance which is balanced by the absorption of oxygen or hydroxyl species onto the surface [52].

### 3.5. Photocatalytic Activity 

The results of photocatalytic activity testing will be discussed separately because of different setups and different light sources and therefore cannot be compared directly. Relative photocatalytic activities of the prepared samples, however, can be discussed and we will do so after the presentation of results.

#### 3.5.1. Liquid-Solid

**Figure 7:**
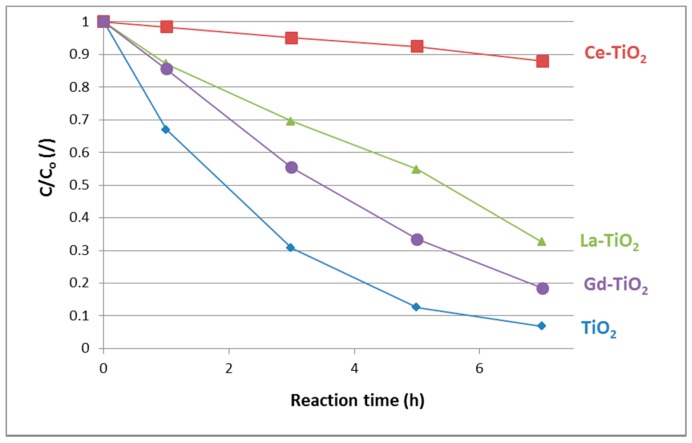
Photocatalytic activity of samples in liquid-solid methylene blue (MB) removal. Sample TiO_2_ shows the highest activity, followed by Gd-TiO_2_, La-TiO_2_, and Ce-TiO_2_.

**Figure 8:**
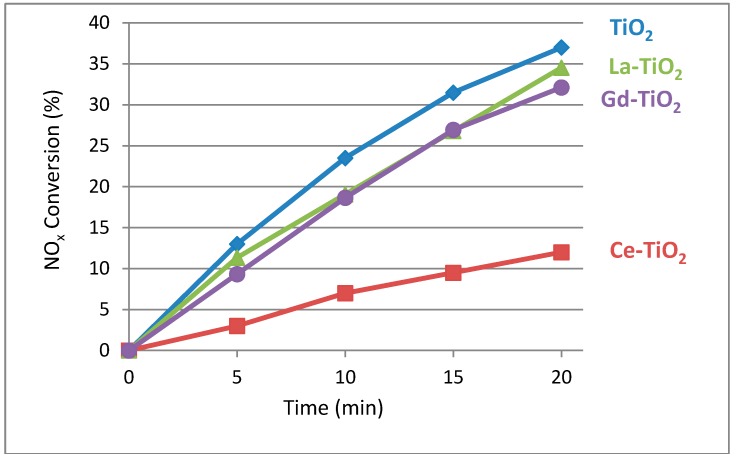
Photocatalytic activity of samples measured by NO_x_ conversion (%) under solar lamp. Again, sample TiO_2_ exhibits the highest activity and Ce-TiO_2_ the lowest. Here, La-TiO_2_ is more active than Gd-TiO_2_, but the difference is minor.

The photolysis of MB under UV irradiation without the presence of photocatalysts was considered negligible due to previous results by one of the authors. The results of liquid-solid photocatalytic testing are depicted in Figure 7. While none of the samples was able to completely decompose MB within 7 h, the differences between the samples are clearly seen. It turns out that the best activity was achieved by the unmodified sample (TiO_2_). Gd-TiO_2_ and La-TiO_2_ were somewhat less active than the unmodified sample, whereas Ce-TiO_2_ showed very low activity, decomposing merely 14% of MB. The acquired data are graphically depicted in Figure 8. Pseudo-first-order reaction kinetic constants were calculated from these data and are presented in Table 3.

#### 3.5.2. Gas-Solid NO_x_ Removal

The samples were tested for their ability to remove inorganic pollutant compounds from the air, namely, NO_x_. The samples were tested for their activity under both UV and VIS irradiation. The initial pseudo-first-order kinetic constant was calculated for the 20 min reaction of NO_x_ abatement. The NO_x_ conversion vs. time is depicted in Figure 8, and the first-order kinetic constant calculation results are presented in Table 4. As before, the Ce-TiO_2_ turned out to be the least active, both under UV and VIS irradiation, despite being the only sample with the light absorption tail reaching out into the visible light region. It is well known that it is not only light absorption that influences photocatalytic activity. In the case of cerium modification, we can see that other detrimental factors outweigh its beneficial visible light absorption. Under UV irradiation, Ce-TiO_2_ is about four times less active than the unmodified sample, while it is inactive under visible light. The activity of the other samples is comparable, the TiO_2_ sample again being the most active one under solar irradiation.

#### 3.5.3. Gas-Solid VOC Removal

The method to measure the photocatalytic activity with regard to VOC oxidation is described in detail above. The results of both Vis and UV + Vis activity are presented in Table 5.

Results under both Vis and UV + Vis once again show that the unmodified sample performed better than the modified ones. Ce-TiO_2_ performed dismally in this test as well, whereas La-TiO_2_ and Gd-TiO_2_ were much better, but still far behind the unmodified sample. Ce-TiO_2_ did not show any activity under VIS light and very limited activity under UV + Vis. TiO_2_ sample activity was on a par with the industrial benchmark P25 in UV + Vis and somewhat outperformed it under Vis light. Higher VIS activity may not be surprising because the synthesized sample is composed of rutile phase only, while P25 is a mixture of anatase and rutile in an approximate 85:15 ratio, favoring anatase [53]. Although some authors claim that the mixture of the two phases is what makes the P25 so active, with rutile acting as antennae into visible light and anatase featuring high surface area plus efficient charge separation with close contact between the phases [54], we have consistently found that pure rutile is on a par with P25 or even outperforms it somewhat. The La-TiO_2_ and Gd-TiO_2_ are a mixture of anatase and rutile, yet their activity in VIS is half that of the unmodified sample and less than half in UV + Vis (see the numbers in Table 5). Whether this is related to modification or to phase content is unclear from the data. The majority of studies in the literature would suggest that the anatase phase is generally more active, although some authors claim that rutile performs better [31,32]. Despite the attempt to improve on photocatalytic activity by modifying RE elements into the TiO_2_ matrix, it turns out that modifying was detrimental rather than beneficial to titania functional properties, both under UV + Vis and Vis.

#### 3.5.4. Comparison between Photocatalytic Activities of Samples in Different Environments

##### Activity under UV Irradiation or Simulated Solar Irradiation (UV + Vis)

**Figure 9:**
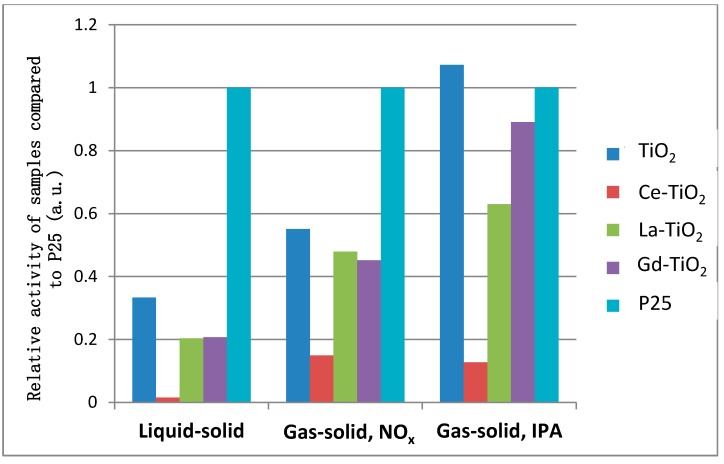
Histograms of photocatalytic activity of samples under UV and solar irradiation. The kinetic constants are normalized to the P25 constant and are presented as percentages of the P25 constant. P25 is therefore by definition always equal to 1.

**Figure 10:**
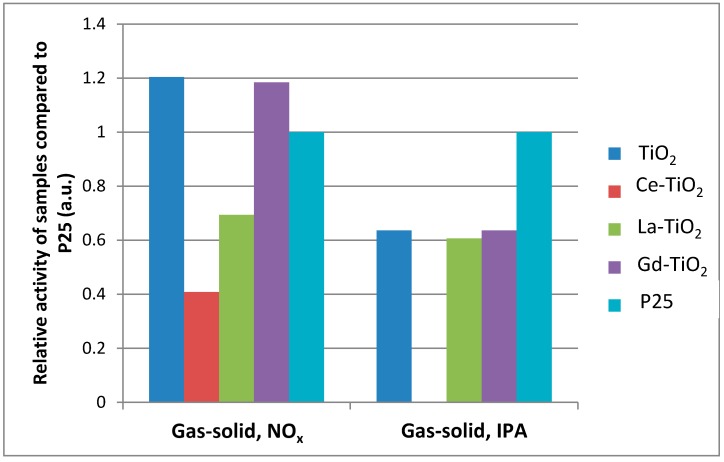
Histograms of photocatalytic activity of samples under visible light irradiation. The kinetic constants are normalized to the P25 constant and are presented as percentages of the P25 constant. P25 is therefore by definition always equal to 1.

While modification proved itself to be detrimental in all cases and with all three RE elements used for modification (Ce, La, and Gd), the diminished photocatalytic activity seems to be specific to the environment and pollutant. As we have seen, modification is also associated with different phase content. Unfortunately, we cannot know the contribution brought in by phase differences and that by modification. Keeping in line with the majority of studies in the literature, one could argue that lower photocatalytic activity was observed despite higher anatase content, not because of it, since anatase is usually perceived as the more active phase. While many authors found that modification with RE might be beneficial to photocatalytic activity, at least for some elements [55,56,57], we have found this not to be the case for any of the three methods used for measuring photocatalytic activity. Figure 9 and Figure 10 compare the different methods of photocatalytic activity measurements. Since this cannot be done directly because of different setups, pollutants, and units of measurement, we compared the activity of synthesized samples to the activity of the industrial benchmark Evonik P25. The histograms in Figure 9 and Figure 10 show the relative activity of samples compared to that of P25 (Y-axis). The relative activity of P25 is by definition equal to one. To calculate the relative activity of our samples, we used data from Table 3, Table 4 and Table 5. The data for P25 activity was taken from Karmaoui et al. [58] for liquid-solid activity and from Tobaldi et al. [39] for gas-solid photocatalytic activity measurement using NO_x_ as a model pollutant. Activity of P25 is also included in Table 3, Table 4 and Table 5, which were used to plot the histograms in Figure 9 and Figure 10. The activity in the gas-solid regime for VOC removal was measured anew. The latter samples were freshly prepared (an important piece of information, since virgin P25 exhibits much higher activity than P25 used repeatedly). The data represented in Figure 9 are given as a ratio of P25 because the setup cannot be directly compared. P25 has therefore been normalized to 1 and others are represented accordingly.

From Figure 10, it is clear that P25 outperforms all but photocatalysts in the liquid-solid regime and in the gas-solid system for NO_x_ abatement. Only sample TiO_2_ had higher activity in IPA removal in a sealed gas-solid system. This sample was also the best performing in other setups. While P25 seems to be universally a good catalyst, the synthesized samples seem relatively better performing in VOC oxidation. We have found that modification with the selected rare earth metals is not favorable to high photocatalytic activity. Cerium modification yields samples with especially low activity, most notable in the liquid-solid environment. Lanthanum and gadolinium modification also negatively affects the samples’ photocatalytic activity, although less so than cerium modification. Modification with these two elements lowers the activity to ~60% of the unmodified sample in the liquid-solid environment and to ~85% in NO_X_ abatement. The effect was similar in these two setups. In VOC removal, however, the effect of the two elements differs.

As previously noted, the unmodified sample outperformed the modified ones in all three of the setups. However, the relative activity of the samples differed among the setups. The Ce-modified sample had the lowest photocatalytic activity in all cases. In the liquid-solid system, it achieved ~5% of the activity of the unmodified sample (Ce-TiO_2_ k_app_ was 0.018 h^−1^, whereas TiO_2_ k_app_ was 0.39 h^−1^). In the gas-solid phase with NO_x_ as a model pollutant, it achieved >25% of the activity of TiO_2_ (Ce-TiO_2_ k_app_ was 0.0066 min^−1^, whereas TiO_2_ k_app_ was 0.0244 min^−1^). In the gas-solid phase with isopropanol as a model pollutant, representing VOCs, the relative activity of Ce-TiO_2_ was ~12% of the unmodified sample (Ce-TiO_2_ k was 103 ppm/h and TiO_2_ k was 886 ppm/h). 

The La-modified sample performed better than Ce-TiO_2_, yet it did not achieve or perform better than the unmodified sample. The La-TiO_2_ sample reached ~60% of activity in the liquid-solid regime. It was relatively most effective in NO_x_ abatement, achieving ~87% of the activity of the unmodified sample (k′_app_ of La-TiO_2_ was 0.0212 h^−1^ and k′_app_ of the unmodified sample was 0.0244 h^−1^). In a gas-solid system for VOC oxidation under simulated solar illumination, the lanthanum-modified sample performed similarly as in the liquid-solid regime. The kinetic constant of La-TiO_2_ was 518 ppm/h and that of TiO_2_ was 886 ppm/h. Relatively speaking, the La-TiO_2_ sample achieved ~60% of the activity of the unmodified sample.

The gadolinium-modified sample yielded similar results of photocatalytic activity to the lanthanum-modified sample in the liquid-solid regime and NO_x_ abatement. However, its photocatalytic activity was considerably higher for VOC degradation, achieving ~83% of the unmodified samples’ activity (kinetic constant being 735 ppm/h for Gd-TiO_2_ and 885 ppm/h for TiO_2_). From the results obtained, we can see that the unmodified sample outperformed other samples, despite consisting of rutile phase only, which is generally considered less active.

##### Activity under Visible Light Irradiation

Photocatalytic activity of the prepared samples under visible light activity was measured only for both of the gas-solid systems. The activity of all the samples was about an order of magnitude lower under simulated solar irradiation. Relative photocatalytic activities of samples under visible light irradiation are presented in Figure 10. As before, activities are normalized to the activity of Evonik P25, which therefore equals 1.

Under visible light irradiation, Ce-TiO_2_ had the lowest activity as well. It was inactive in NO_x_ abatement and achieved about a third of the activity of the unmodified sample in the VOC degradation system. Unmodified sample and La- and Gd-modified samples had similar activity in the NO_x_ abatement system. However, in the VOC removal system, the La-modified sample had lower activity than the Gdmodified and unmodified sample. The activity of the samples under visible light for VOC removal is as follows: TiO_2_ > Gd-TiO_2_ > La-TiO_2_ > Ce-TiO_2_.

At the present time, it is unclear why the relative activity of the samples is so different when used in different photocatalytic activity setups. Further research may show that a specific photocatalyst has to be engineered for the optimal degradation of selected pollutants. P25 seems to be the most versatile photocatalyst, yet it still falls short in VOC degradation.

## 4. Conclusions

In our work we modified TiO_2_ using selected rare earth elements (Ce, La, and Gd) to understand their effect on its structural, optical, morphological, and photocatalytic properties. The addition of the selected elements significantly influenced the phase composition of the prepared photocatalysts. While the unmodified sample contained pure rutile phase, all the modified samples were a mixture of rutile and anatase (La-TiO_2_ and Gd-TiO_2_) or rutile, anatase, and brookite (Ce-TiO_2_). Thus, the addition of rare earth elements to hydrothermal processing slowed anatase-to-rutile and brookite-to-rutile transformation. Cerium had the strongest impact on the transformation. The reason for the delayed anatase-to-rutile transformation was attributed to grain-boundary pinning by the rare earth elements gathering on the surface of anatase and brookite particles [45].

A significant visible light absorption tail reaching up to 585 nm wavelength was detected only in sample Ce-TiO_2_, whereas samples La-TiO_2_ and Gd-TiO_2_ did not show a definitive difference in light absorption compared to the unmodified sample. The long tail into visible light absorption of Ce-TiO_2_ indicates the presence of CeO_2_ on the surface of TiO_2_.

Three different photocatalytic activity measurement setups were compared using P25 as reference material. The order of photocatalytic isopropanol oxidation under simulated solar light (UV + Vis) was TiO_2_ > (P25) > Gd-TiO_2_ > La-TiO_2_ > Ce-TiO_2_. Under Vis illumination, the order was TiO_2_ ≈ Gd-TiO_2_ > (P25) > La-TiO_2_ > Ce-TiO_2_. In gas-solid NO_x_ abatement under UV irradiation, all samples had lower activity than P25, the most active being the unmodified sample, which reached ~55% of P25 activity level. Less active were samples La-TiO_2,_ Gd-TiO_2_, and Ce-TiO_2_, reaching 48%, 45%, and 15% of P25 activity, respectively. Under Vis irradiation, Ce-TiO_2_ did not show detectable activity, despite having shown light absorption well into the visible light spectrum, up to ~585 nm wavelength. Other samples showed similar photocatalytic activity of approximately 60% of P25 activity. In the liquid-solid regime under UV irradiation, the relative activity of synthesized samples was lower. The unmodified sample reached 33% of P25 activity, La-TiO_2_ and Gd-TiO_2_ reached ~20% of P25 activity, and Ce-TiO_2_ barely showed any activity at all. We attributed the reason for the unmodified sample’s high activity to the large Ti^3+^/Ti^4+^ ratio and the high proportion of hydroxyl oxygen compared to the modified samples, which had a lower Ti^3+^/Ti^4+^ ratio and less hydroxyl-bound oxygen. The Ti^3+^/Ti^4+^ ratio and the oxygen forms in the samples were analyzed using the XPS method. Oxygen in modified samples was mostly lattice-bound oxygen. As for why the relative activity of the samples was different in the photocatalytic activity measurement regimes used, that question is still under investigation.

## Figures and Tables

**Table 1:** Rietveld agreement factors and phase composition of the unmodified and modified samples.

Sample No. of Variables		Agreement Factors			Phase Composition		
		Rf^2^ (%)	R_wp_ (%)	χ^2^	Anatase (wt.%)	Rutile (wt.%)	Brookite (wt.%)
TiO_2_	12	5.68	8.59	2.117	/	100	/
Ce-TiO_2_	22	5.83	5.95	1.999	56.1 ± 0.3	35.6 ± 0.2	8.3 ± 0.7
La-TiO_2_	15	5.49	7.27	2.389	26.0 ± 0.4	74.0 ± 0.1	/
Gd-TiO_2_	19	4.66	7.29	2.427	25.4 ± 0.5	74.6 ± 0.1	/

**Table 2:** Band gap of the samples calculated by taking the maximum of the first derivative of reflectance (dR/dλ) vs. wavelength (λ) as the band gap of a sample. Sample TiO_2_ *E_g_* is the smallest, which is not surprising, since it consists of rutile. Mixed phase samples’ *E_g_* is higher. The *E_g_* of Ce-TiO_2_ is the highest.

Sample	1st Derivative Maximum	
	Wavelength (nm)	*E_g_* (eV)
TiO_2_	401	3.09
Ce-TiO_2_	395	3.14
La-TiO_2_	400	3.10
Gd-TiO_2_	399	3.11

**Table 3:** Pseudo-first-order reaction kinetic constants for the synthesized samples that were calculated on the basis of points from Figure 7.

Sample	k′_app_ (h^−1^)
TiO_2_	0.390
Ce-TiO_2_	0.018
La-TiO_2_	0.238
Gd-TiO_2_	0.242
P25	1.17

**Table 4:** Pseudo-first-order reaction constant under the solar lamp (k′_app_ solar) and under visible light irradiation (k′_app_ Vis) calculated on the basis of data points from Figure 8. P25 activity is added for comparison.

Sample	Photocatalytic Activity	
	k′_app_ Solar (min^−1^)	k′_app_ VIS (min^−1^)
TiO_2_	0.0244	0.0042
Ce-TiO_2_	0.0066	0.0000
La-TiO_2_	0.0212	0.0040
Gd-TiO_2_	0.0200	0.0042
P25	0.04427	0.0066

**Table 5:** Photocatalytic activity of the samples in gas-solid regime as measured by VOC removal. Their activity was measured under both solar lamp (UV + Vis activity) and Vis illumination only (wavelengths approximately 400 nm). Unmodified TiO_2_ shows the highest activity, followed by Gd-TiO_2_, La-TiO_2_, and Ce-TiO_2_ the lowest.

Sample	Photocatalytic Activity	
	Vis (ppm/h)	UV + Vis (ppm/h)
TiO_2_	59	885
Ce-TiO_2_	20	105
La-TiO_2_	34	520
Gd-TiO_2_	58	735
P25	49	825
